# *Geobacteraceae* are important members of mercury-methylating microbial communities of sediments impacted by waste water releases

**DOI:** 10.1038/s41396-017-0007-7

**Published:** 2018-01-10

**Authors:** Andrea G. Bravo, Jakob Zopfi, Moritz Buck, Jingying Xu, Stefan Bertilsson, Jeffra K. Schaefer, John Poté, Claudia Cosio

**Affiliations:** 1 Limnology and Science for Life Laboratory, Uppsala University, Uppsala, SE-75236 Sweden; 2 Aquatic and Stable Isotope Biogeochemistry, University of Basel, Basel, CH-4056 Switzerland; 3 Environmental Sciences, Rutgers University, New Brunswick, NJ 08901 USA; 4 Environmental Biogeochemistry and Ecotoxicology, University of Geneva, Geneva, CH-1205 Switzerland; 5 Present Address: Unité Stress Environnementaux et BIOSurveillance des Milieux Aquatiques UMR-I 02 (SEBIO), Université de Reims Champagne Ardenne, Reims, F-51687 France

## Abstract

Microbial mercury (Hg) methylation in sediments can result in bioaccumulation of the neurotoxin methylmercury (MMHg) in aquatic food webs. Recently, the discovery of the gene *hgcA*, required for Hg methylation, revealed that the diversity of Hg methylators is much broader than previously thought. However, little is known about the identity of Hg-methylating microbial organisms and the environmental factors controlling their activity and distribution in lakes. Here, we combined high-throughput sequencing of 16S rRNA and *hgcA* genes with the chemical characterization of sediments impacted by a waste water treatment plant that releases significant amounts of organic matter and iron. Our results highlight that the ferruginous geochemical conditions prevailing at 1–2 cm depth are conducive to MMHg formation and that the Hg-methylating guild is composed of iron and sulfur-transforming bacteria, syntrophs, and methanogens. *Deltaproteobacteria*, notably *Geobacteraceae*, dominated the *hgcA* carrying communities, while sulfate reducers constituted only a minor component, despite being considered the main Hg methylators in many anoxic aquatic environments. Because iron is widely applied in waste water treatment, the importance of *Geobacteraceae* for Hg methylation and the complexity of Hg-methylating communities reported here are likely to occur worldwide in sediments impacted by waste water treatment plant discharges and in iron-rich sediments in general.

## Introduction

Methylmercury (MMHg) is a neurotoxin that is biomagnified in aquatic food webs with fish consumption as a primary route for human MMHg exposure [1]. In aquatic ecosystems, methylation of mercury (Hg) to MMHg is carried out by microorganisms that are usually present at oxic-anoxic boundaries of sediments [2], water columns [3] and settling particles [4]. Studies on sediments have implicated sulfate-reducing bacteria (SRB) as the main contributors to MMHg formation, as inhibition of SRB with molybdate often decreases MMHg production rates [5–9]. Accordingly, many studies have aimed to identify the SRB strains responsible for Hg methylation by testing their Hg-methylating potential in pure cultures [10, 11], or by determining correlations between Hg methylation and the presence of SRB markers in sediments [12, 13] or water column [5]. Few studies have however linked Hg methylation to iron-reducing bacteria (FeRB) [14, 15], or methanogenic archaea [16]. The identification of the genes (*hgcAB*) required for Hg methylation [17] led to the discovery that the diversity of Hg methylators was much broader and the process more complex than previously thought [18]. Notably, the ability to methylate Hg has been identified within new phyla (*Firmicutes* and *Chloroflexi*) featuring also fermentative and acetogenic metabolisms [17–19]. The discovery of genetic markers for Hg-methylation also opened the door to more efficient, standardized, and detailed monitoring of Hg methylators in the environment. Accordingly, microbial Hg-methylating communities have recently been described in wetlands and paddy soils and included methanogens, SRB, FeRB, and syntrophs [19–22]. Hg-methylating microbial communities in lakes, on the other hand, remain poorly investigated with regards to the types of microorganisms involved and the factors influencing their activities, except that MMHg formation is determined by the molecular composition of organic matter (OM) [23].

Waste production can have a strong impact on the chemical and biological characteristics of freshwaters [24, 25]. As an example, treated waste water from the City of Lausanne is discharged into Lake Geneva (Switzerland) via an outlet pipe located in Vidy Bay. The dephosphatation treatment, based on the addition of ferric chloride to the waste water, causes enhanced inputs of Fe into the bay [24, 26]. Moreover, the current volume of sewage exceeds the capacity of the waste water treatment plant (WWTP), requiring transient releases of partially untreated water, which contributes to the enrichment of the sediments with OM and various trace metals [27]. Among them Hg is of major concern because of its toxicity and the high ambient concentrations (e.g., refs. [2, 25]). Previous studies have shown that the areas affected by the WWTP discharge are hotspots for MMHg formation and concentration [2]. In particular, it was found that sediments enriched in OM with prevailing ferruginous conditions (i.e., high concentrations of dissolved Fe^II^ and no free sulfide in the porewater) had the highest Hg methylation rates found in the bay [2]. Furthermore, sediment amendment experiments revealed that inhibition of dissimilatory sulfate reduction with molybdate led to increased Fe-reduction rates and enhanced Hg methylation. It was thus concluded that microorganisms other than SRB were responsible for Hg methylation [2], but the identity of the Hg-methylating microorganisms and thus the pathways by which MMHg was formed were not elucidated.

Building on this earlier discovery and recognizing the potential global implications of enhanced Hg methylation rates in WWTP-influenced sediments, the aim of this study was to provide a mechanistic understanding of MMHg formation in Fe-rich freshwater sediments. For this purpose, we linked sediment geochemistry and Hg speciation with parallel analysis of the genes coding for 16S rRNA and *hgcA*. In addition, the functional potential of the microbial communities was assessed by qPCR quantification of specific genes involved in the biogeochemical cycling of carbon, sulfur and iron. This is the first study in WWTP-impacted sediments exploring the diversity of microorganisms involved in Hg methylation.

## Material and methods

### Study site and sampling

The present study was conducted in the Vidy Bay of Lake Geneva, one of the largest freshwater reservoirs in Europe. Samples were collected at a site close to the WWTP outlet pipe (CP, 46°30'52.89” N, 6°35'21.25” E) based on previous studies showing a strong impact of the WWTP on MMHg formation [2]. Three replicate sediment cores (A, B, and C) were collected in August 2010 as detailed in the Supplementary Information.

### Chemical analysis

Total carbon, organic carbon (C_org_), inorganic carbon (C_inorg;_ samples exposed twice to 5 % H_3_PO_4_ overnight) and nitrogen (N_tot_) in sediments were measured with a CHN elemental analyzer (Perkin Elmer 2400 series II). OM content was determined by loss on ignition (LOI) [28].

Metals were extracted from freeze-dried sediment with 2 N HNO_3_ at 100 °C for 12 h. Total Fe and S (Fe_tot_; S_tot_) were measured by ICP-AOS (iCAP-6300 Duo Thermo Scientific). A detailed description of mercury speciation and quantification can be found elsewhere [2]. Briefly, sediment total Hg (Hg_tot_) was quantified using an Advanced Mercury Analyser (254 Altec). MMHg in sediments was extracted by HNO_3_ leaching/CH_2_Cl_2_ extraction and measured by ethylation onto Tenex traps followed by GC separation [29]. Hg_tot_ in porewater was measured by CV-AFS [30]. MMHg in porewater was measured by species-specific isotope dilution and GC-ICPMS [31].

Porewater concentrations of SO_4_^2−^ and NO_3_^−^ were analyzed by ion-chromatography (Dionex ICS-3000) with an Ion-Pac_AS19 column. Poorly crystalline Fe^III^ -oxides in the sediment were extracted with 0.5 M HCl, reduced to Fe^II^ with 1 M hydroxylamine hydrochloride and subsequently quantified with the ferrozine method [32]. Elemental sulfur (S^0^) was extracted from wet sediment with methanol and subsequently analyzed by RP-HPLC and UV detection at 265 nm [33].

### DNA extraction and bacterial community composition: 16S rRNA gene

DNA was extracted in triplicate from freeze-dried sediments with the Power Soil DNA Extraction kit (Mo-Bio) as previously described [34]. PCR primers 341 F (5′-CCTACGGGNGGCWGCAG-3′) and 805 R (5′-GACTACHVGGGTATCTAATCC-3′) were used for amplification of the 16S rRNA gene from most bacteria [35, 36]. The resulting PCR products were used as template in an additional 10-cycle amplification with sample-specific barcoded primers according to Sinclair et al. [37]. See Supplementary Table 1 and Supplementary Information for details.

Amplicon sequencing data (Illumina MiSeq pair-end 300 bp) were preprocessed using FastQC [38] and the illumitag pipeline as described in Sinclair et al. [37]. Chimeric sequences were removed and reads were grouped into Operational Taxonomic Units (OTU) clustering at 97 % sequence identity. The taxonomical annotation of the OTUs was subsequently performed by CREST using the SILVA database [37]. Data were not rarefied to avoid overlooking rare members of the communities that may be more sensitive to the environmental gradients of interest [39]. Sequence data have been deposited in the European Nucleotide Archive (http://www.ebi.ac.uk/ena) under the accession number PRJEB20838.

### Hg methylator community composition: *hgcA*

Primers targeting *hgcA* sequences were adopted from Schaefer et al. [22] and modified to include secondary priming-sequences for a second stage PCR where sample-specific barcodes and Illumina sequencing adaptors were added. For this purpose, the forward primer hgcA_261 F (5′-CGGCATCAAYGTCTGGTGYGC-3′) and the reverse primer hgcA_912 R (5′-GGTGTAGGGGGTGCAGCCSGTRWARKT-3′) with barcode adaptors, were first used to amplify the *hgcA* gene from each sample. For the second PCR, each sample was individually barcoded with unique combinations of forward and reverse primers (Supplementary Table 2). For details on the amplification strategy, see Supplementary Fig. 1. Second PCR amplicons were then purified using Agencourt AMPure XP (Beckman Coulter, USA), quantified using the PicoGreen kit (Invitrogen) and subsequently pooled in equal proportions. Amplicons were sequenced using the Illumina MiSeq instrument and pair-end 300 bp mode. Due to the length of the PCR product, only the forward read sequence was used for downstream data-analysis. Bad quality reads were trimmed with SICKLE [40], and left-over adapters and primers were removed with CUTADAPT [41]. Reads were truncated, dereplicated and singletons removed with USEARCH version 8.0 [42]. The obtained set of reads was then clustered using cd-hit-est with a 60 % similarity threshold [43]. The original quality-controlled reads were finally mapped to the representative sequences of the obtained clusters to generate a count table using the USEARCH software. The database used for annotation of the sequences is based on the sequences used by Podar et al. [19], additionally a Hidden Markov Model (HMM) based on these sequences was made using HMMER [44] and subsequently used to mine *δ-Proteobacteria* from the IMG database of JGI. The Sequences where curated and the taxonomy homogenized. Analogous to the 16S rRNA gene diversity analysis, data were not rarefied. More detailed information on the construction of *hgcA* gene libraries and phylogenetic analyses is given in Supplementary Information. Sequence data can be found in the European Nucleotide Archive under the accession number PRJEB20838.

### Quantitative PCR

Quantitative PCR (qPCR) of marker genes (16S rRNA*, dsrA, GCS, mcrA*, and *merA*) for key functional groups involved in carbon, sulfur and iron cycling was performed in 10 µL total reaction volumes with primer pairs listed in Table S3 using an Eco cycler (Illumina, San Diego, California) and the Kapa SYBR Fast qPCR kit (Kapa Biosystems, Wilmington, Massachusetts), as described in Bravo et al. [45]. All reactions were performed according to the supplier’s protocol with 40 amplification cycles and real-time data acquired during the annealing step of each cycle. Standard curves and no-template controls were included in triplicate for each reaction and the target copy number per sample was calculated. The quality of standard curve and melting curves were tested with qpcR package (www.dr-spiess.de/qpcR.html; [46, 47]) in the freely available software environment R version 3.1.0 (http://www.r-project.org/). See Supplementary Information for more details.

### Statistical analysis

Pearson correlations were calculated between chemical compounds using centered and reduced values in Excel (Microsoft, Redmond, WA, USA). Rarefaction curves (Supplementary Fig. 2) were plotted using R. Barplots showing relative abundances of major taxa were built with the RAM package (http://cran.r-project.org/package = RAM, [48]).

## Results

### Chemical analyses

**Fig. 1:**
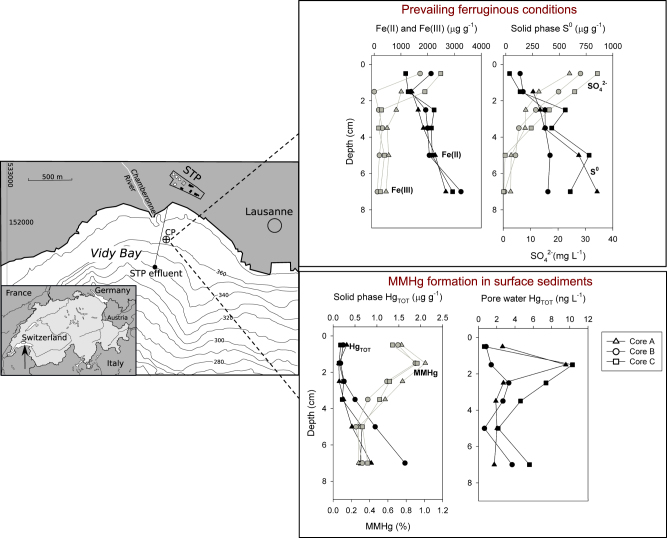
Map of the study area and chemical characterization of solid phases and porewater in three sediment cores collected at site CP near the outlet pipe of the sewage treatment plant (STP). Complete data are given in Supplementary Table 4.

The geochemical composition of the 18 sediment samples features gradual changes with depth, but little variation among the 3 replicate sediment cores (Fig. 1; Supplementary Table 4). The decrease of SO_4_^2−^ together with increasing Fe^II^ and S^0^ concentrations with depth indicates that H_2_S produced by SRB is titrated out from the porewater by Fe^III^ (Fig. 1, Supplementary Table 4). As a consequence, porewater sulfide concentrations remain low throughout the whole sediment core [2]. Carbon, OM and nitrogen varied only little with depth, with C_org_ representing about 40 % of C_tot_, OM ranging from 5.5 to 7.2 %, and C_org_/N-ratio ranging from 5.5 to 11.3, indicative of fresh OM of predominantly microbial and algal origin. Generally, the geochemical conditions are very similar to an earlier study conducted at the same site [2].

Hg_tot_ increased with depth from 0.13–1.67 µg g^−1^ d.w. (range of the three core replicates) to 1.73–5.58 µg g^−1^ d.w. at the deepest layer (Fig. 1). These values are 4- to 50-times higher than the natural background of Hg_tot_ in Lake Geneva (0.03 mg kg^−1^) [49]. MMHg concentrations were generally higher in the surface layer (0–1 cm: 1.1–2.3 ng g^−1^) and in deeper layer (6–8 cm: 2.4–5.2 ng g^−1^) than at intermediate depth (Fig. 1, Supplementary Table 4). The proportion of MMHg to Hg_tot_ was the highest at 1–2 cm (0.9–1.1 %) and decreased with depth to values between 0.28 % and 0.38 %. These Hg_tot_ values and MMHg percentages are in line with analyses performed previously, but fall within the lower range of Hg concentrations reported for Vidy Bay [49, 50]. Pearson's correlation analysis revealed that MMHg concentrations were positively correlated to C_org_, S_tot_, Fe^II^ and Hg_tot_ (Figure S3). The proportion of Hg_tot_ as MMHg was also correlated with porewater SO_4_^2−^ concentration, and inversely to the sedimentary S^0^ content (Supplementary Fig. 3). Overall, our results confirm the important role of S, Fe and Hg for the geochemistry in sediments of Vidy Bay [2].

### Bacterial community composition: 16S *rRNA* gene

**Fig. 2:**
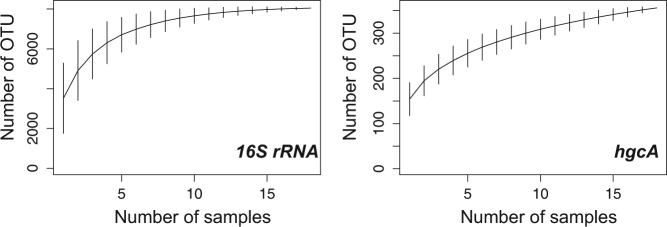
Number of OTU’s detected as function of the number of collected sediment samples from Vidy Bay. Plots were made with the *specaccum* function of the Vegan package in R. The OTU clustering was done at 97 and at 88 % similarity levels for 16S rRNA gene and *hgcA* gene sequences, respectively.

**Fig. 3:**
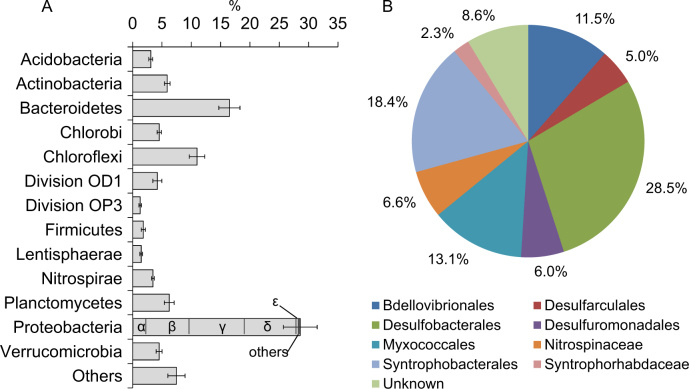
Phylogenetic distribution of bacterial 16S rRNA gene sequences in the combined Vidy Bay sediment dataset: **A**. Relative abundance of major phyla and **B** classes within the δ-*Proteobacteria*. Presented data are average percentages (±SE) of reads from the 18 collected samples (6 depths in 3 cores). Categories representing >1 % are shown.

The composition and diversity of the combined bacterial community was investigated by sequencing of 16S rRNA amplicons. Close to 524’000 sequences (between 13’133 and 59’205 per sample) were clustered into OTUs at three different identity levels; 80 % roughly corresponding to the phylum level, 95 % corresponding to genus-level, and 97 % to species-level, the latter with 8044 OTUs identified. The overall coverage of the sediment community is reflected in the combined richness detected for random subsets of analyzed samples, where the logarithmic shape indicated that most of the species richness occurring in the sediments was covered in the combined dataset (Fig. 2). Overall the bacterial community composition was quite uniform across replicates and samples (Fig. 3, Supplementary Fig. 4), and showed only a slight change in diversity with depth at the OTU level (Supplementary Fig. 5).

Phylogenetic analysis of 16S rRNA gene sequences identified *Proteobacteria* as the dominant phylum in terms of richness and relative abundance, represented by 1’605 OTUs or 28.6 ± 1.6 % of the reads, followed by *Bacteroidetes* (629 OTUs, 16.5 ± 2.4 %), and *Chloroflexi* (615 OTUs, 11 ± 2.7 %; Fig. 3). Within the *Proteobacteria*, δ-*Proteobacteria* (869 OTUs) were abundant and accounted for 31 % of the *Proteobacteria* reads. *Firmicutes*, mostly represented by *Clostridiales*, contributed 1.9 % of the total reads, but had representatives among the 50 most abundant OTUs in the total data set (Supplementary Table 5). Members of the *Syntrophobacterales*, including, e.g., *Syntrophus*, *Syntrophorhabdus*, and *Syntrophobacteraceae*, were also detected. Moreover, several lineages involved in Fe-transformation (e.g., *Acidithiobacillus*, *Geobacter*, *Geothermobacter*, and *Shewanella*; [51]) and sulfur cycling (e.g., *Desulfobulbaceae*, *Desulfobacteraceae*, etc.) were identified. Among the genera known to host representatives that carry *hgcAB* genes [18, 19], we identified *Geobacter*, *Syntrophus*, *Syntrophorhabdus*, *Desulfobulbus*, *Desulfomonile*, *Desulfovibrio*, *Desulfomicrobium*, unknown *Ruminococcaceae* genus, and *Syntrophomonadaceae*. In particular, *Geobacter*, *Syntrophus*, and *Syntrophorhabdus* represented 0.15, 0.29, and 0.21 % of total 16S rRNA reads, respectively.

### Quantification of functional genes

Quantification of total bacterial abundance by qPCR detected between 1.4 × 10^7^ and 6.4 × 10^8^ 16S rRNA gene copies g^−1^ of wet sediment (Supplementary Table 6). Abundance of 16S rRNA gene was highest in the uppermost sediment layers and decreased slightly with depth. Analyses of selected functional genes revealed wide occurrences of SRB (*dsrA*), *Geobacteraceae* (*GCS*) and methanogens (*mcrA*) (Supplementary Table 6). The abundance of SRB, *Geobacteraceae* and *merA*—encoding the mercuric reductase—slightly decreased with depth. Methanogens reached their highest representation in the deepest sediment layers, where the main electron acceptors Fe^III^ and sulfate were depleted (Fig. 1).

### A closer look at the Hg-methylating community through *hgcA* gene analysis

**Fig. 4:**
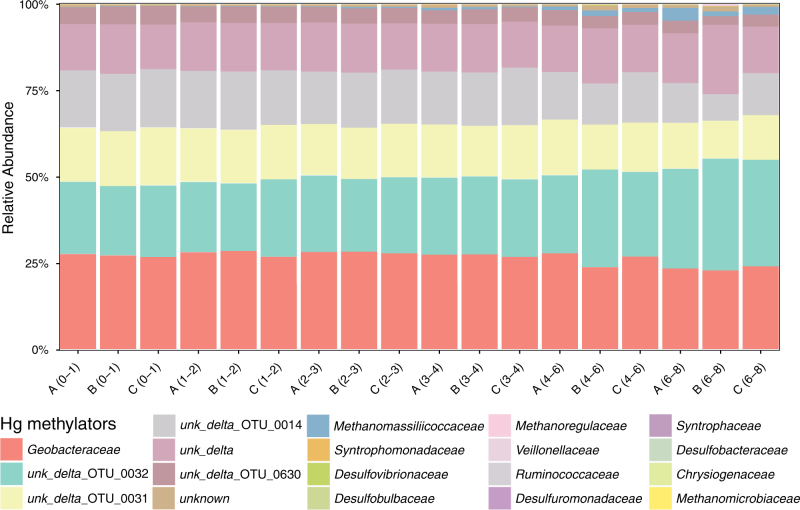
Relative abundance of Hg-methylating families carrying *hgcA* in Vidy Bay sediments. “OTU_0032”, “OTU_0031”, “OTU_0014”, and “OTU_0630”, all abundant members of the Hg-methylating community, were annotated as unknown *δ-Proteobacteria*. Numbers in parentheses represent the sediment depth interval in centimeters.

In addition to the phylogenetic community analysis, the diversity of Hg-methylating microorganisms was for the first time assessed by high-throughput sequencing of the *hgcA* gene. As for 16S rRNA, community rarefaction indicated that most of the Hg methylator diversity targeted by these primers was captured (Fig. 2). Analogous to 16S rRNA, Hg-methylating community composition was also quite uniform across replicates and samples with only minor changes with depth (Fig. 4, Supplementary Fig. 5). The *hgcA* gene data set consisted of altogether 741’890 reads ranging from 22’522 to 62’442 reads per sample. A total of 356 OTUs were identified at 88 % identity level, 325 of which were affiliated with *Bacteria*, 25 with *Archaea*, while the affiliation of 6 OTUs could not be resolved. Among the bacterial *hgcA* OTU’s, 264 were annotated as *Proteobacteria*, 41 as *Firmicutes* while 20 could not be linked to any specific phylum. In terms of their relative abundance, *Bacteria* represented 99 % of the reads, while *Archaea* represented 0.8 % of the reads.

All proteobacterial *hgcA* reads grouped with δ-*Proteobacteria*, with 27 % of the reads being *Desulfuromonadales* (153 OTUs), 71 % were from unknown δ-*Proteobacteria* (92 OTUs) and 0.1 % represented other *δ*-*Proteobacteria*, including *Desulfovibrionales, Desulfobacterales* and *Syntrophobacterales* (Figure S6). The 17 most abundant *hgcA* OTUs contributed 92 % of all reads and were all affiliated with *δ-Proteobacteria*. In particular, the three most abundant OTUs in terms of read counts (“OTU_0032”, “OTU_0031” and “OTU_0014”) were annotated as unknown *δ-Proteobacteria* and represented 22.8, 14.9, and 15.1 % of the reads, respectively (Fig. 4). The fourth most abundant OTU was affiliated with *Geobacter* spp. and contributed 14.7 % of reads. In addition, an unknown *δ-Proteobacteria* “OTU_0630” represented 4.4 % of the reads (Fig. 4). At higher taxonomic resolution, 99 % of the *Desulfuromonadales* reads were affiliated with *Geobacteraceae* (147 OTUs, 27 % of all *hgcA* reads). The two most abundant archaeal *hgcA* OTUs were affiliated with *Methanomassiliicoccus* spp. and each represented about 0.3 % of total *hgcA* reads. These OTUs were 20–112 times more abundant in the 4–6 cm and 6–8 cm of sediment than in 0–1 cm and 1–2 cm layers (Fig. 4). Hg-methylating *Firmicutes* were also detected (0.2 % of reads). In particular, a *Dethiobacter* sp. (*Syntrophomonadaceae*) represented 0.1 % of reads, whereas an *Ethanoligenens* sp. (*Ruminococcaceae*) accounted for 0.02 % of reads.

**Fig. 5:**
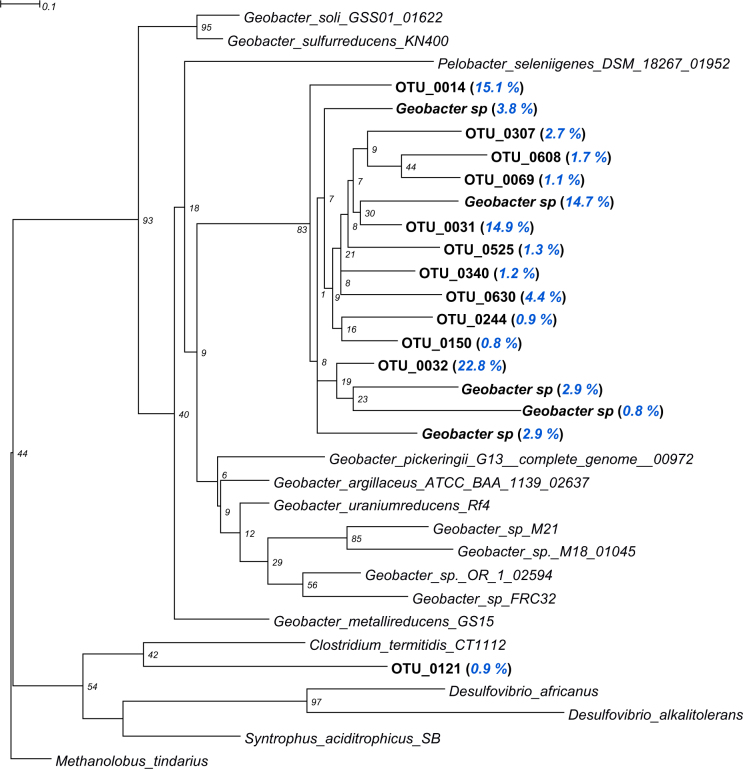
Phylogenetic distribution of the 17 most abundant *δ-Proteobacteria* of Vidy Bay sediments (names in bold) based on *hgcA* sequences. The tree was generated using RAxML (version 8.2.4) with the PROTGAMMLG model and the autoMR to choose the number of necessary bootstraps (750).

Phylogenetic analysis suggested that the most abundant Hg-methylating OTUs (“OTU_0032”, “OTU_0031” and “OTU_0014”) of Vidy Bay sediments were phylogenetically related to *Geobacter* species (Fig. 5). Therefore, when summing the reads of “OTU_0032”, “OTU_0031”, “OTU_0014”, and “OTU_0630” with those of identified *Geobacteraceae*, our data suggested that *Desulfuromonadales* could contribute to more than 85 % of the reads encountered (Fig. 4, Supplementary Fig. 6). Hence unknown Hg-methylating δ-*Proteobacteria* and unknown Hg-methylating bacteria combined, would account for less than 15 % of the reads (Supplementary Fig. 6). One OTU (“OTU_0121”), representing 0.9 % of reads and initially annotated as *δ-Proteobacteria*, was more closely related to *Clostridium* spp. according to the phylogenetic analysis.

## Discussion

### WWTP effluents foster specific geochemical conditions that favor Hg methylation processes

Effluents of the WWTP with Fe as dephosphatation agent are a known source of Hg [49] and create geochemical conditions favoring Hg methylation [2]. Here the highest proportion of Hg_tot_ as MMHg was observed at 1–2 cm depth (Fig. 1, Table S4). The ferruginous conditions of Vidy Bay sediments, i.e. high porewater Fe^2+^ concentrations but virtually no dissolved sulfide available to bind Hg (Fig. 1), most likely keep Hg bound to OM [52] and thus available for methylation. Also, degradation of specific organic compounds, or reductive dissolution of Fe^III^(oxy)hydroxides under anoxic conditions, likely trigger the release of Hg, as indicated by porewater Hg_tot_ peaking at this specific depth and accordingly enhance net MMHg formation in this layer. Considering that the abundance of functional genes (Supplementary Table 6), bacterial communities (16S rRNA, Figure S4) and the Hg-methylating communities (*hgcA*, Fig. 4, Supplementary Fig. 6) showed only minor variation over depth across all studied sediment profiles, our results highlight that geochemical conditions prevailing at 1–2 cm depth control MMHg formation. Thus the effluents from the WWTP might not only exert a strong influence on the redox states of Fe and S but also on Hg speciation and cycling.

### *Geobacteraceae*: dominant members of the Hg-methylating guild in ferruginous sediments

By combined analysis of 16S rRNA and *hgcA* sequences, we identified microorganisms potentially involved in MMHg production. Here the observed richness and abundance imply that FeRB but also fermenters and other hitherto unknown Hg methylators are members of the Hg-methylating microbial community in sediments affected by OM- and Fe-rich WWTP effluents. However, both the 16S rRNA gene and *hgcA* gene analyses showed that *δ-Proteobacteria*, and in particular *Desulfuromonadales*, were abundant. Recent studies of *hgcA* diversity in wetlands and rice paddies have concluded that *δ-Proteobacteria* dominate Hg-methylating microbial communities [20–22] and this seems to hold also for the freshwater sediments studied here. Although the primers used in these studies were designed based on genomic DNA from a small subset of the known Hg-methylating microorganisms, most of them *δ-Proteobacteria*, our results demonstrate that *hgcA* sequencing was also successful in detecting some *Archaea* (Fig. 4) and members of other bacteria phyla such as *Firmicutes*. Among *δ-Proteobacteria*, the richness and the abundance of *Geobacter* spp. in the *hgcA* data set pointed to their importance for Hg methylation in the studied sediments. Only a few previous studies have demonstrated that Hg methylation in sediments can occur under Fe-reducing conditions with FeRB mediating the process [2, 14, 15]. Instead, SRB have historically been identified as the principal Hg methylators in aquatic systems [9, 12, 13, 53, 54]. Although we identified several SRB by 16S rRNA gene sequencing—with quantification of *drsA* gene confirming that SRB are widely present in these sediments—the abundance of identified SRB (e.g., *Desulfovibrio* spp.) in the *hgcA* diversity analyses was significantly smaller than for fermenters (e.g., *Ruminococcaceae* and *Syntrophomonadaceae*) and FeRB (*Geobacteraceae*). The latter observation is in line with previous cultivation-based estimates suggesting FeRB to be three orders of magnitude more abundant than SRB in these sediments and that Fe^III^ reduction rates was positively correlated to Hg methylation rates [2].

### A geomicrobiological model for coupled Fe, S cycling and MMHg formation in sediments affected by WWTP discharges

We propose that FeRB play a central role in both Hg methylation and anaerobic OM degradation in these sediments. Indeed, *Geobacter* spp., known to inhabit sediments and sludge [55], can reduce Fe^III^ or S^0^ but also perform interspecies electron transfer to S^0^-reducers or methanogens [56]. Syntrophic bacteria, however, have been recently identified as putatively important Hg methylators [21], and have also been detected in sediments adjacent to the WWTP discharge pipe outlet [57]. Accordingly, one known syntrophic lineage, *Smithella* spp. (*Syntrophaceae*, 17 reads) was identified in the current *hgcA* analyses. Some lineages detected in Vidy Bay sediments, such as *Syntrophobacteraceae* (3574 reads*)* and *Syntrophaceae* (5805 reads), might degrade OM in syntrophic association with H_2_ consuming organisms, such as FeRB, SRB or methanogens [58, 59]. In this context, indirect effects of non-Hg-methylating microorganisms on MMHg formation should also be considered. For example methanogens could maintain the activity of syntrophic Hg methylators, especially in deeper sediment layers where Fe^III^ and SO_4_^2−^ are depleted [60]. In short, it is likely that *Geobacter* sp get enriched by the continuous import of Fe^III^ to the sediment surface and continuous reoxidation of Fe^II^ at its surface through mixing. In deeper sediment layers, these microbes likely survive through alternative metabolic processes such as S^0^ reduction or syntrophic oxidation of OM [60]. Considering our results here and previous research carried out in Vidy Bay [2], we propose a geomicrobiological model (Figure S7) where Fe reacts with sulfide (either produced by SRB through sulfate reduction or liberated from biomass through anaerobic degradation of amino acids) according to: 2FeOOH + HS^−^ + 5 H^+^ ⇒ 2Fe^2+^ + S^0^ + 4H_2_O. As a result, anoxic conditions prevail in these sediments while sulfide is low and Fe^2+^ and S^0^ are high. Elemental sulfur in these sediments might then be recycled by S^0^-reduction performed by e.g., *Geobacter* or *Dethiobacter* [61], which are both identified Hg methylators [19]. Hence, Fe^III^ might act as a direct electron acceptor for *Geobacter*, but also as an sulfide “scrubber” according to the stoichiometry above and as previously suggested for freshwater sediments (Haveman et al., 2008; [62]). These results provide novel understanding on the role of *Geobacter* in Hg methylation processes linked to Fe and S cycling in a low-sulfate freshwater environment.

## Conclusions

Previous efforts to identify Hg methylators have mainly been limited to functional characterization of cultured isolates and inferences made about their close relatives based on phylogenetic markers such as the 16S rRNA gene. A more recent alternative strategy has been to probe directly *hgcA* genes using target amplification, cloning and sequencing. Here, combination of high-throughput sequencing approach with molecular barcoding and amplification protocols identified Hg-methylating microbial communities in freshwater sediments. Our results reveal the coexistence of a wide abundance of fermenters, secondary fermenters (e.g. syntrophs), SRB, FeRB, and methanogens in this Hg-contaminated freshwater lake ecosystem and point to the significant contribution of syntrophs, fermenters, Fe and S reducers to the diversity and abundance of the Hg-methylating community in the sediments rich in OM and Fe. Moreover, known SRB Hg methylators constituted relatively minor groups compared to the total Hg-methylating and SRB community, supporting the idea that sediments affected by WWTP effluents are in this regard different from most other anoxic aquatic settings previously studied. Our results suggest that geochemical conditions rather than the composition of the resident microbiota dictate net MMHg formation. Since Fe is widely used in waste water treatment across the globe, the importance of *Geobacteraceae* for Hg methylation and the geochemical conditions reported here are likely relevant for WWTP recipients worldwide.

## Electronic supplementary material

Supporting information
