# Supplementary material for: Geobacteraceae are important members of mercury-methylating microbial communities of sediments impacted by waste water releases
Source: ISME J. 2018 Jan 10;12(3):802–12. doi: 10.1038/s41396-017-0007-7 (PMC5864163; doi:10.1038/s41396-017-0007-7)
Supplement: Supplementary file 1 — Supporting information [file 41396_2017_7_MOESM1_ESM.docx]

***Supplementary Information***

***Geobacteraceae* are important members of mercury-methylating microbial communities of sediments impacted by wastewater releases**

Andrea G. Bravo, Jakob Zopfi, Moritz Buck, Jingying Xu, Stefan Bertilsson, Jeffra K. Schaefer, John Poté, Claudia Cosio*

Number of pages (including cover sheet and references): 18

This file includes:

- Supplementary Material and Methods
- 7 Supplementary Figures
- 6 Supplementary Tables

*corresponding author: claudia.cosio@univ-reims.fr

**SI - Materials and Methods**

**Sampling**

Three replicate sediments cores were collected in August 2010 and immediately brought to the laboratory, where they were kept cooled and upright with overlying water from the sampling site. Further processing was done within 8 h. The cores were opened in a glove box under N_2_ atmosphere to avoid oxidation of reduced chemical species, and sectioned into one cm-thick slices for the upper 4 cm, followed by 2 cm-thick slices down to 8 cm depth. The porewater was collected by centrifugation (4’000 *g*) and passed through a 0.45 μm membrane filter (Sterivex, Millipore) under N_2_ atmosphere. Aliquots were immediately acidified by HCl addition to 0.5 % v/v final concentration for Hg determination. Sediment solids were immediately freeze-dried for metal analysis and DNA extraction, while aliquots were also frozen for analysis of Fe and S^0^.

**DNA extraction and Quantitative PCR**

DNA was extracted in triplicate from freeze-dried homogenized sediments as described above. DNA in the extracts was quantified by UV absorbance and the concentration adjusted to 5 ng μl^−1^ with water containing molecular biology grade bovine serum albumin (Fluka) at a concentration of 3 μg ul^−1^ and heated for 5 min at 90 °C to bind PCR inhibiting substances (Regier *et al.*, 2012).

To prepare standards, specific primers were used on extracted DNA from sediments or DNA from pure cultures with previously described primer pairs (Table S1). PCR amplicons were ligated into a pGEM-T Easy vector (Promega, Madison, WI, USA) and cloned in *Escherichia coli* DH5α. Clones containing the target gene insert were sequenced and the most abundant variant was used for plasmid DNA extraction. After measuring the DNA concentration (Qubit, Invitrogen), the purified plasmid was diluted serially in 10×-steps and subjected to qPCR in triplicate to generate an external standard curve.

The quality of standard and melting curves was tested with the qpcR package (www.dr-spiess.de/qpcR.html; Tichopad *et al.*, 2003; Ritz and Spiess, 2008) in R (http://www.r-project.org/). Standard curves had an amplification efficiency ranging between 90 to 98% and a R^2^ > 0.980. In addition the absence of primer dimers or other non-specific amplicons in these reactions were further confirmed by visualizing the PCR samples on an agarose gel.

**DNA amplification and sequencing of 16S rRNA genes**

PCR primers 341F (5’-CCTACGGGNGGCWGCAG-3’) and 805R (5’-GACTACHVGGGTATCTAATCC-3’) were used for amplification of the 16S rRNA gene from most bacteria. The resulting PCR products were diluted hundredfold in sterile Q-grade water. One microliter of the diluted PCR triplicates were pooled and used as template in an additional 10-cycle amplification with sample-specific barcoded primers according to Sinclair *et al.* (2015). All PCR amplifications were carried out in 20 µl reactions using 1 U Phusion high fidelity DNA polymerase (NEB, UK), 0.25 µM primers, 200 µM dNTP mix, and 0.4 µg bovine serum albumin (NEB, UK). Amplicons were gel purified with the Qiagen gel purification kit according to manufacturer instructions (Qiagen, Germany) and quantified with a fluorescence-based DNA quantitation kit (PicoGreen, Invitrogen). Uniquely barcoded amplicons from 50 samples were pooled in equimolar amounts to have roughly similar sequencing depth for each sample (see below for PCR product purification procedures).

Amplicon sequencing was carried out at the SNP/SEQ SciLifeLab facility hosted by Uppsala University, using Illumina MiSeq in pair-end 300 bp read length mode. The data were preprocessed with version 2.1.13 of the Illumina instrument control software. Further statistics were produced using FastQC (Andrews, 2012). Sequences were processed using the illumitag pipeline as described previously (Sinclair *et al.*, 2015). Chimeric sequences were removed and reads were grouped into Operational Taxonomic Units (OTU) clustering at 97 % sequence identity. The taxonomical annotation of the OTUs was subsequently performed by CREST using the SILVA database following Sinclair et al. (2015). Data were not rarefied to avoid overlooking rare members of the communities that may be more sensitive to the environmental gradients of interest (McMurdie and Holmes, 2014).

**Construction of *hgcA* gene libraries and phylogenetic analyses**

Primers targeting *hgcA* sequences were adopted from Schaefer *et al.* (2014) and modified to include secondary priming-sequences for a second stage PCR where sample-specific barcodes and Illumina sequencing adaptors were added. For this purpose, the forward primer hgcA_261F (CGGCATCAAYGTCTGGTGYGC) and the reverse primer hgcA_912R (GGTGTAGGGGGTGCAGCCSGTRWARKT) with barcode adaptors, were first used to separately PCR amplify the *hgcA* gene from each sample in 50 μl volumes with 1× Phusion GC Buffer, 0.2 mM dNTP mix, 5 % DMSO, 0.1 μM of each primer adaptor, 7 μg μl^-1^ of bovine serum albumin (NEB, UK), 4 μl extracted DNA template, and 1.0 U Phusion high fidelity DNA polymerase (NEB, UK) for an initial denaturation of 2 min at 98 °C followed by 35 cycles (10 s at 96 °C, 30 s 56.5 °C and 45 s at 72 °C), and a final extension at 72 °C for 7 min. For the second PCR, each sample was individually barcoded with unique combinations of forward and reverse primers (Table S2). For details on the amplification strategy, see supplementary Figure S1. Reactions were conducted in 20 μl volumes using 1× Q5 reaction buffer, 0.2 mM dNTP mix, 0.1 μM barcoded primers, purified first PCR amplicons and 1.0 U Q5 high fidelity DNA polymerase (NEB, UK) for an initial denaturation step of 30 s at 98 °C followed by 18 cycles (10 s at 98 °C, 30 s 66 °C and 30 s at 72 °C), and a final extension at 72 °C for 2 min. Second PCR amplicons were then purified using Agencourt AMPure XP (Beckman Coulter, USA), quantified using the PicoGreen kit (Invitrogen) and subsequently pooled in equal proportions. Amplicons were sequenced at the SNP/SEQ SciLifeLab facility hosted by Uppsala University using the Illumina MiSeq instrument and pair-end 300 bp mode.

Due to the length of the PCR-product only the forward half of the amplicon was used for data-analysis. Bad quality reads, adapters and primers were trimmed with SICKLE and CUTADAPT (Joshi and Fass, 2011; Martin, 2011). Version 8.0 of the USEARCH software was used to truncate ('-fastx_truncate'), dereplicate (-derep_prefix), sort and remove singletons ('-sort_by_size -minsize 2') (Edgar, 2013). The obtained set of reads was then clustered using cd-hit-est with a 60% similarity threshold (Fu *et al.*, 2012). The original cleaned reads are finally mapped to the representative sequences of the obtained clusters to generate a count table, using USEARCH software again ('-usearch_global'). The database used for the annotation of the sequences is based on the sequences previously used (Podar *et al.*, 2015), additionally a Hidden Markov Model (HMM) based on these was made with HMMER (Eddy, 2011) and used to mine *δ-Proteobacteria* from the Integrated Microbial Genomes (IMG) database of the Joint Genome Institute (JGI).

The Sequences where adequately curated and taxonomy homogenized using taxtastic [https://github.com/fhcrc/taxtastic] and R-package taxize (Chamberlain and Szöcs, 2013). The obtained protein sequences were aligned with MUSCLE (Edgar, 2004) (version 3.8.1551). The alignment was trimmed to the size of the amplicon, and a tree was generated using RAxML (Stamatakis, 2014) (version 8.2.4) - with the PROTGAMMLG model and the autoMR to choose the number of necessary bootstrap resamplings (n = 750) - was used to generate a tree, from which paralogs were manually removed. This tree and the corresponding alignment were used to generate a reference package for PPLACER (Matsen *et al.*, 2010). The guppy tool of PPLACER was then used to classify the sequences with a likelihood threshold of 0.8.

**Figure S1** Sample treatment procedures for amplification of 16S rRNA and *hgcA* genes (modified from Sinclair et al., 2015).

**
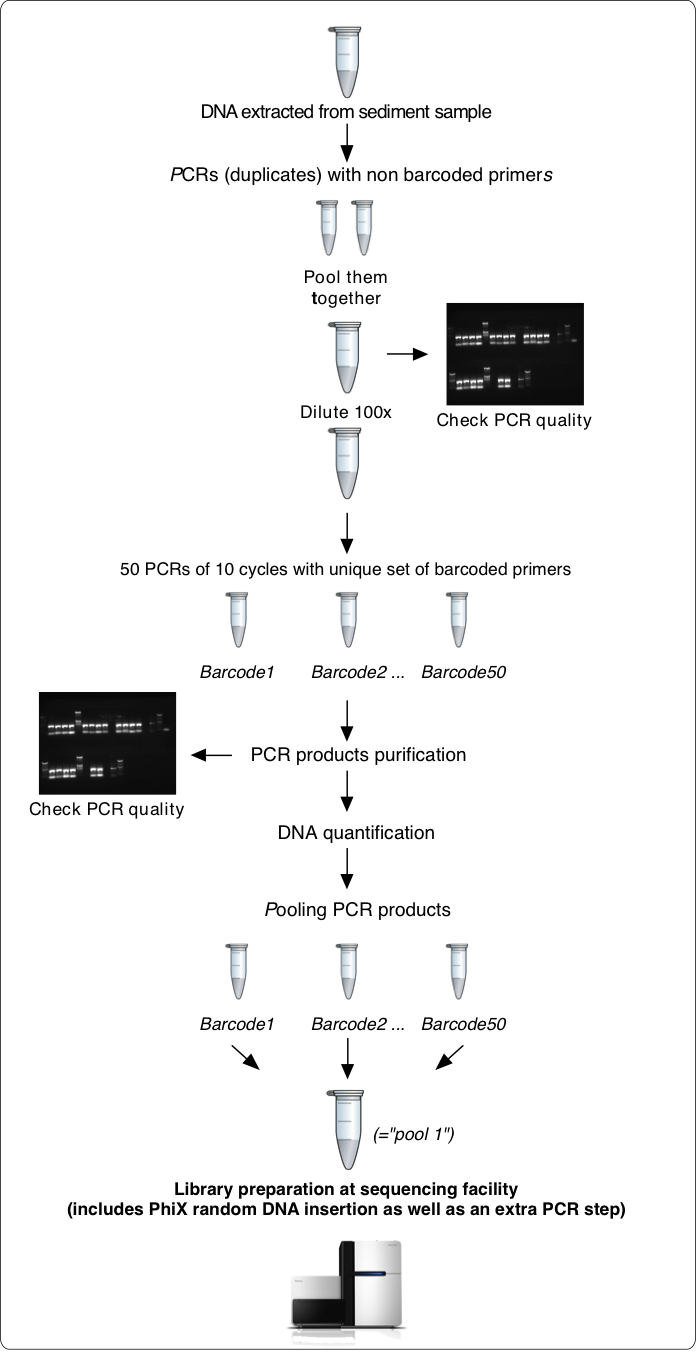
**

**Figure S2.** Rarefaction curves of 16S rRNA (blue) and *hgcA* (pink) genes for the 18 samples of sediments collected at site CP in the Vidy Bay. The x-axis represents the number of samples while the y-axis represents the number of detected OTU's. Numbers in parenthesis represent the depth interval in centimeters.

**Figure S3**: Selected parameters showing significant Pearson correlation score between ancillary parameters in solid phase and pore water (pw) in sediments of Vidy Bay (** *p* < 0.01, **p* < 0.05). Significant correlations appear in bold.

|  |
| --- |

**Figure S4.** Relative abundance of the 20 most abundant bacteria classes (16S rRNA gene) in different depth intervals of 3 replicate sediment cores collected close to the WWTP outlet pipe. Numbers in parenthesis represent the depth interval in centimeters.

**Figure S5.** Non-metric multidimensional scaling (NMDS) plot visualizing the composition of the bacterial community based on Bray-Curtis distances of the 16S rRNA (2D stress = 0.09) and *hgcA* (2D stress = 0.1) data.

**Figure S6** Relative abundance of Hg-methylators (16S rRNA gene) in different depth intervals of 3 replicate sediment cores collected close to the WWTP outlet pipe. Phylogenetic analyses (Figure 5 of the MS) suggest that unknown *Deltaproteobacteria* OTU0032, OTU0031, OTU0014 and OTU630 are closely related to *Desulfuromonadales.* Numbers in parenthesis represent the depth interval in centimeters.

**Figure S7**. Geomicrobiological model of coupled Fe/S cycling and MMHg production in the ferruginous freshwater sediments of Vidy Bay, Lake Geneva. Left: Vertical concentration profiles of Fe-oxides, porewater sulfate and percentage of MMHg of total Hg, in arbitrary units. Center/right: Major biotic (regular arrows) and abiotic (dashed arrows) processes based on geochemical and sequence data. Fe and S are ultimately buried in the sediment as FeS/FeS_2_. Hg may be sequestered into these solid phases, reduced to Hg^0^ via mineral surface mediated redox reaction or methylated by microorganisms. MMHg-producing microorganisms involved in OM degradation, Fe^III^-reduction, sulfate- and sulfur-reduction that have been identified by their *hgcA* sequence are given in bold next to the corresponding process. Names given in regular text stand for known Hg-methylating taxa that have been detected by 16S rRNA gene sequencing only. FeRB: Fe^III^-reducing bacteria; SRB: sulfate- reducing bacteria.

**Table S1** Barcoded primers used for 16 rRNA gene

| Sample | Code in pool | Forward barcode | Reverse barcode |
| --- | --- | --- | --- |
| A0-1 | A0 | GACTGAT | TGACACT |
| A1-2 | A1 | GACATCA | TGACTCA |
| A2-3 | A2 | TAGAGAG | TGTGCTA |
| A3-4 | A3 | GAGTCAT | TGTGTCA |
| A4-6 | A4 | GAGAGAT | TGTCGTA |
| A6-8 | A6 | GCTATCA | TGTCACA |
| B0-1 | B0 | GCTACTA | TACTAGC |
| B1-2 | B1 | GCTCATA | TCGCATA |
| B2-3 | B2 | GCATACT | TCGTAGA |
| B3-4 | B3 | GCATCAT | TCAGCTA |
| B4-6 | B4 | TAGAGCA | TCAGAGA |
| B6-8 | B6 | TATCACG | ACTGAGA |
| C0-1 | C0 | TACGTCA | ACTGACT |
| C1-2 | C1 | ACATCAG | ACTGATC |
| C2-3 | C2 | ACACTGT | ACTGTGT |
| C3-4 | C3 | ACACACT | ACTCGAT |
| C4-6 | C4 | ACACAGA | ACTCAGT |
| C6-8 | C6 | ACGATGT | ACTCTGA |

**Table S2** Barcoded primers used for *hgcA* gene

| Sample | Code in pool | Forward barcode | Reverse barcode |
| --- | --- | --- | --- |
| A0-1 | A0 | ACTGCATA | CTAGTACG |
| A1-2 | A1 | ACTGCATA | TTCTGCCT |
| A2-3 | A2 | ACTGCATA | GCTCAGGA |
| A3-4 | A3 | ACTGCATA | AGGAGTCC |
| A4-6 | A4 | ACTGCATA | CATGCCTA |
| A6-8 | A6 | ACTGCATA | GTAGAGAG |
| B0-1 | B0 | ACTGCATA | CCTCTCTG |
| B1-2 | B1 | ACTGCATA | AGCGTAGC |
| B2-3 | B2 | ACTGCATA | CAGCCTCG |
| B3-4 | B3 | ACTGCATA | TGCCTCTT |
| B4-6 | B4 | ACTGCATA | TCCTCTAC |
| B6-8 | B6 | ACTGCATA | GGTATAAG |
| C0-1 | C0 | ACTGCATA | CAGCTAGA |
| C1-2 | C1 | ACTGCATA | CCATAGCA |
| C2-3 | C2 | ACTGCATA | GGTATAGC |
| C3-4 | C3 | ACTGCATA | GGTTATGC |
| C4-6 | C4 | ACTGCATA | TAGGCAAG |
| C6-8 | C6 | ACTGCATA | TTGTCCAT |

**Table S3:** Primers used to characterize the distribution of microbial functional groups by qPCR (Bravo *et al.*, 2016).

| **Gene** | **Primer sequence 5’**→ **3’** | **Process/function** | **Target** |
| --- | --- | --- | --- |
| 16S rRNA | ACTCCTACGGGAGGCAGCAG | Universal primer | Bacteria (El Fantroussi *et al.*, 1999) |
|  | ATTACCGCGGCTGCTGG |  |  |
| *dsrA* | ACSCACTGGAAGCACG | Reduction of sulfite to sulfide:  SO_3_^2-^ → H_2_S | Sulfate reducing bacteria (Ben-Dov *et al.*, 2007) |
|  | GTGTAGCAGTTACCGCA |  |  |
| *gltA* | TTCCGYGGYAWGACMATTCC | Citrate synthase gene of *Geobacteraceae* (GCS) | *Geobacteraceae* (Bond *et al.*, 2005) |
|  | TCCCARGTGATGTTBGCCAWGC |  |  |
| *mcrA* | GGTGGTGTMGGDTTCACMCARTA | Methane generation:  CH_3_-S-CoM → CH_4_ | Methanogens (Steinberg and Regan, 2008) |
|  | CGTTCATBGCGTAGTTVGGRTAGT |  |  |
| *merA* | CGATCCGCAAGTGGCIACBGT | Hg reduction:  Hg^II^ → Hg^0^ | Hg resistance (Schaefer *et al.*, 2004) |
|  | ACCATCGTCAGRTARGGRAAVA |  |  |

**Table S4:** Chemical characterization of the three sediment cores collected at site CP near the outlet pipe of the WWTP.

|  |  |  |  | | | Sediments | | | | | | | | | |  | Pore water | | | | |
| --- | --- | --- | --- | --- | --- | --- | --- | --- | --- | --- | --- | --- | --- | --- | --- | --- | --- | --- | --- | --- | --- |
| Core | Layer |  | C_tot_ | N_tot_ | C_org_/N | | OM | Fe^II^ | Fe^III^ | S^0^ | Fe_tot_ | S_tot_ | Hg_tot_ | MMHg | MMHg |  | Hg_tot_ | MMHg | MMHg | NO_3_^-^ | SO_4_^2-^ |
|  | cm |  | % | % | % | | % | µg·g^-1^ ww | µg·g^-1^ ww | µg·g^-1^ ww | mg·g^-1^ dw | mg·g^-1^ dw | µg·g^-1^ dw | ng·g^-1^ dw | % |  | ng·L^-1^ | ng·L^-1^ | % | mg·L^-1^ | mg·L^-1^ |
| **A** | **0-1** |  | 5.2 | 0.3 | 5.5 | | 7.2 | nd | nd | nd | 14.0 | 1.7 | 0.31 | 2.30 | 0.75 |  | 2.63 | <dl |  | 0.37 | 24.2 |
|  | **1-2** |  | 4.9 | 0.3 | 6.5 | | 5.9 | 1404.8 | 1009.3 | 254.6 | 12.8 | 2.0 | 0.13 | 1.30 | 1.01 |  | 9.57 | 0.79 | 8.3 | 0.57 | 13.0 |
|  | **2-3** |  | 4.1 | 0.2 | 1.0 | | 5.4 | 1642.6 | 823.2 | 320.5 | 14.8 | 2.4 | 0.14 | 1.10 | 0.76 |  | 2.73 | <dl |  | 0.36 | 8.2 |
|  | **3-4** |  | 4.6 | 0.3 | 7.2 | | 5.8 | 1839.0 | 481.8 | 360.3 | 11.0 | 2.4 | 0.24 | 1.40 | 0.57 |  | 1.90 | <dl |  | 0.38 | 8.0 |
|  | **4-6** |  | 4.8 | 0.3 | 6.4 | | 6.3 | 2279.7 | 539.3 | 681.9 | 14.9 | 3.6 | 0.43 | 1.20 | 0.28 |  | 2.03 | <dl |  | 0.61 | 2.9 |
|  | **6-8** |  | 4.7 | 0.3 | 6.1 | | 6.5 | 2675.8 | 440.6 | 853.0 | 13.2 | 4.1 | 0.87 | 2.40 | 0.28 |  | 1.73 | <dl |  | 0.36 | 2.7 |
| **B** | **0-1** |  | 5.1 | 0.3 | 7.0 | | 6.7 | 2123.9 | 1714.7 | 133.2 | 14.0 | 1.5 | 0.23 | 1.60 | 0.71 |  | 0.84 | 0.34 | 40.4 | 0.32 | 28.2 |
|  | **1-2** |  | 4.4 | 0.3 | 7.0 | | 5.7 | 1359.3 | 0 | 160.8 | 15.9 | 1.9 | 0.17 | 1.50 | 0.90 |  | 1.39 | <dl |  | 0.49 | 20.2 |
|  | **2-3** |  | 4.7 | 0.3 | 7.6 | | 6.1 | 1922.9 | 164.8 | 365.8 | 16.7 | 2.9 | 0.25 | 1.50 | 0.59 |  | 3.32 | <dl |  | 0.46 | 11.9 |
|  | **3-4** |  | 5.0 | 0.3 | 7.4 | | 6.4 | 1994.8 | 305.0 | 367.5 | 12.7 | 2.6 | 0.50 | 1.90 | 0.38 |  | 2.68 | <dl |  | 0.76 | 5.7 |
|  | **4-6** |  | 4.7 | 0.3 | 7.3 | | 6.2 | 2059.5 | 191.2 | 412.5 | 14.8 | 5.0 | 0.96 | 2.40 | 0.25 |  | 0.66 | 0.22 | 32.8 | 0.24 | 4.5 |
|  | **6-8** |  | 5.2 | 0.3 | 11.3 | | 8.7 | 3245.0 | 114.7 | 393.1 | 13.0 | 7.1 | 1.64 | 5.20 | 0.32 |  | 3.68 | <dl |  | 0.32 | 0.5 |
| **C** | **0-1** |  | 5.0 | 0.3 | 7.4 | | 6.4 | 1174.3 | 2488.3 | 35.2 | 15.3 | 1.4 | 0.17 | 1.10 | 0.65 |  | 0.64 | 0.32 | 50.9 | 0.41 | 34.4 |
|  | **1-2** |  | 4.1 | 0.2 | 7.8 | | 5.5 | 1270.6 | 1897.1 | 133.1 | 14.2 | 1.7 | 0.14 | 1.30 | 0.92 |  | 10.30 | 0.52 | 5.0 | 0.42 | 26.1 |
|  | **2-3** |  | 5.0 | 0.3 | 8.0 | | 6.2 | 2240.3 | 268.4 | 556.5 | 14.7 | 2.7 | 0.24 | 1.50 | 0.62 |  | 7.40 | <dl |  | 0.39 | 16.8 |
|  | **3-4** |  | 4.8 | 0.3 | 7.4 | | 6.2 | 2140.1 | 162.8 | 429.2 | 15.5 | 3.2 | 0.21 | 1.10 | 0.51 |  | 4.61 | <dl |  | 0.48 | 10.2 |
|  | **4-6** |  | 5.3 | 0.3 | 8.1 | | 6.2 | 2165.0 | 382.1 | 778.6 | 11.8 | 3.2 | 0.66 | 2.10 | 0.32 |  | 2.16 | <dl |  | 0.52 | 0.7 |
|  | **6-8** |  | 5.1 | 0.3 | 7.2 | | 6.1 | 2932.1 | 230.9 | 602.5 | 13.5 | 4.1 | 0.62 | 2.40 | 0.38 |  | 5.59 | <dl |  | 0.21 | 0.2 |

C_org_: organic carbon, C_tot_: total carbon; dl: detection limit, OM: organic matter; ww: wet weight; dw: dry weight.

**Table S5** The 10 most abundant bacterial taxa according to 16S rRNA analysis in different depth sections of 3 replicate sediment cores (A, B, C) collected at site CP. Numbers in parenthesis represent the depth interval in centimeters.

| **A(0-1)** | **A(1-2)** | **A(2-3)** | **A(3-4)** | **A(4-6)** | **A(6-8)** |
| --- | --- | --- | --- | --- | --- |
| WCHB1-69 | WCHB1-69 | WCHB1-69 | WCHB1-69 | division OD1 | vadinHA17 |
| Acidimicrobiaceae | division OD1 | division OD1 | division OD1 | vadinHA17 | division OD1 |
| division OD1 | vadinHA17 | vadinHA17 | vadinHA17 | WCHB1-69 | Anaerolineae |
| PeM15 | OPB35 soil group | OPB35 soil group | Anaerolineae | Anaerolineae | WCHB1-69 |
| SB-1 | Anaerolineae | Anaerolineae | OPB35 soil group | Nitrospiraceae | Ignavibacteriales |
| KD4-96 | Nitrospiraceae | Rhodocyclaceae | Nitrospiraceae | Rhodocyclaceae | Nitrospiraceae |
| Crenothrix | Ignavibacterium | Nitrospiraceae | Ignavibacterium | OPB35 soil group | Anaerolineaceae |
| OPB35 soil group | Rhodocyclaceae | Ignavibacterium | Ignavibacteriales | Ignavibacteriales | endosymbionts (Desulfobacteraceae) |
| vadinHA17 | Crenothrix | Crenothrix | Rhodocyclaceae | Acidimicrobiaceae | Ignavibacterium |
| Rhodocyclaceae | Anaerolineaceae | Anaerolineaceae | Anaerolineaceae | Sinobacteraceae | OPB35 soil group |
| **B(0-1)** | **B(1-2)** | **B(2-3)** | **B(3-4)** | **B(4-6)** | **B(6-8)** |
| division OD1 | WCHB1-69 | division OD1 | division OD1 | vadinHA17 | vadinHA17 |
| WCHB1-69 | division OD1 | WCHB1-69 | vadinHA17 | division OD1 | division OD1 |
| vadinHA17 | Acidimicrobiaceae | vadinHA17 | WCHB1-69 | Anaerolineae | Anaerolineaceae |
| OPB35 soil group | Candidatus Rhabdochlamydia | Anaerolineae | Anaerolineae | WCHB1-69 | Anaerolineae |
| Anaerolineae | vadinHA17 | OPB35 soil group | OPB35 soil group | Ignavibacteriales | division OP8 |
| Rhodocyclaceae | Rhodocyclaceae | Rhodocyclaceae | Rhodocyclaceae | OPB35 soil group | Caldisericum |
| Anaerolineaceae | PeM15 | Anaerolineaceae | Anaerolineaceae | Sinobacteraceae | Clostridium |
| Candidatus Rhabdochlamydia | KD4-96 | Ignavibacterium | Crenothrix | Crenothrix | division OP3 |
| Nitrospiraceae | Sinobacteraceae | Nitrospiraceae | Sinobacteraceae | Anaerolineaceae | Ignavibacteriales |
| Sinobacteraceae | Nitrospira (genus) | Sinobacteraceae | Nitrospiraceae | Rhodocyclaceae | SB-5 |
| **C(0-1)** | **C(1-2)** | **C(2-3)** | **C(3-4)** | **C(4-6)** | **C(6-8)** |
| division OD1 | division OD1 | division OD1 | division OD1 | vadinHA17 | vadinHA17 |
| WCHB1-69 | WCHB1-69 | vadinHA17 | WCHB1-69 | division OD1 | division OD1 |
| OPB35 soil group | OPB35 soil group | WCHB1-69 | vadinHA17 | WCHB1-69 | Anaerolineae |
| vadinHA17 | vadinHA17 | Anaerolineae | OPB35 soil group | OPB35 soil group | WCHB1-69 |
| Ignavibacterium | Anaerolineae | OPB35 soil group | Anaerolineae | Anaerolineae | Ignavibacteriales |
| Anaerolineae | Anaerolineaceae | Ignavibacterium | Ignavibacterium | Nitrospiraceae | OPB35 soil group |
| SB-1 | Nitrospiraceae | Anaerolineaceae | Crenothrix | Ignavibacteriales | Rhodocyclaceae |
| Flexibacter | Ignavibacteriales | Nitrospiraceae | Rhodocyclaceae | Rhodocyclaceae | Nitrospiraceae |
| Comamonadaceae | Sinobacteraceae | Rhodocyclaceae | Nitrospiraceae | Anaerolineaceae | Anaerolineaceae |
| Rhodocyclaceae | Ignavibacterium | Ignavibacteriales | Sinobacteraceae | Ignavibacterium | Ignavibacterium |

**Table S6:** Gene abundances determined by qPCR (mean ± SD, n=3) in the three sediment cores collected at site CP.

| **Core** | **Layer** |  | ***16S*** | | |  | ***dsrA*** | | |  | ***GCS*** | | |  | ***merA*** | | |  | | ***mcrA*** | | |
| --- | --- | --- | --- | --- | --- | --- | --- | --- | --- | --- | --- | --- | --- | --- | --- | --- | --- | --- | --- | --- | --- | --- |
|  | cm |  | Log copy·g^-1^ sediment (w.w.) | | | | | | | | | | | | | | | | | | | |
| **A** | **0-1** |  | 8.6 | ± | 0.1 |  | 6.1 | ± | 0.0 |  | 6.6 | ± | 0.0 |  | 6.6 | ± | 0.1 | |  | 4.1 | ± | 0.0 |
|  | **1-2** |  | 8.2 | ± | 0.1 |  | 6.2 | ± | 0.0 |  | 6.9 | ± | 0.2 |  | 6.4 | ± | 0.2 | |  | 4.4 | ± | 0.1 |
|  | **2-3** |  | 7.9 | ± | 0.1 |  | 5.9 | ± | 0.1 |  | 6.3 | ± | 0.1 |  | 6.2 | ± | 0.0 | |  | 4.1 | ± | 0.0 |
|  | **3-4** |  | 7.9 | ± | 0.0 |  | 6.1 | ± | 0.1 |  | 6.5 | ± | 0.0 |  | 6.2 | ± | 0.1 | |  | 4.8 | ± | 0.1 |
|  | **4-6** |  | 7.9 | ± | 0.0 |  | 6.3 | ± | 0.0 |  | 6.4 | ± | 0.0 |  | 6.2 | ± | 0.1 | |  | 4.9 | ± | 0.1 |
|  | **6-8** |  | 7.6 | ± | 0.1 |  | 5.6 | ± | 0.1 |  | 6.1 | ± | 0.1 |  | 5.9 | ± | 0.0 | |  | 5.0 | ± | 0.1 |
| **B** | **0-1** |  | 8.8 | ± | 0.2 |  | 7.0 | ± | 0.0 |  | 7.4 | ± | 0.0 |  | 7.2 | ± | 0.2 | |  | 3.6 | ± | 0.0 |
|  | **1-2** |  | 8.4 | ± | 0.1 |  | 6.4 | ± | 0.1 |  | 6.9 | ± | 0.1 |  | 6.6 | ± | 0.1 | |  | 4.4 | ± | 0.1 |
|  | **2-3** |  | 8.4 | ± | 0.0 |  | 6.4 | ± | 0.1 |  | 6.8 | ± | 0.1 |  | 6.5 | ± | 0.2 | |  | 5.2 | ± | 0.1 |
|  | **3-4** |  | 8.4 | ± | 0.3 |  | 6.6 | ± | 0.0 |  | 6.6 | ± | 0.1 |  | 6.3 | ± | 0.1 | |  | 5.1 | ± | 0.1 |
|  | **4-6** |  | 8.2 | ± | 0.2 |  | 6.4 | ± | 0.1 |  | 6.6 | ± | 0.0 |  | 6.4 | ± | 0.1 | |  | 5.4 | ± | 0.1 |
|  | **6-8** |  | 7.1 | ± | 0.4 |  | 5.3 | ± | 0.0 |  | 5.9 | ± | 0.0 |  | 5.9 | ± | 0.0 | |  | 6.5 | ± | 0.1 |
| **C** | **0-1** |  | 8.1 | ± | 0.0 |  | 5.9 | ± | 0.1 |  | 6.4 | ± | 0.0 |  | 6.4 | ± | 0.0 | |  | 3.8 | ± | 0.0 |
|  | **1-2** |  | 7.8 | ± | 0.3 |  | 5.8 | ± | 0.0 |  | 6.2 | ± | 0.0 |  | 6.1 | ± | 0.0 | |  | 4.1 | ± | 0.0 |
|  | **2-3** |  | 7.9 | ± | 0.2 |  | 5.9 | ± | 0.0 |  | 6.3 | ± | 0.0 |  | 6.1 | ± | 0.1 | |  | 4.5 | ± | 0.1 |
|  | **3-4** |  | 8.0 | ± | 0.1 |  | 6.0 | ± | 0.0 |  | 6.3 | ± | 0.1 |  | 6.2 | ± | 0.0 | |  | 4.5 | ± | 0.1 |
|  | **4-6** |  | 8.1 | ± | 0.0 |  | 6.1 | ± | 0.0 |  | 6.4 | ± | 0.0 |  | 6.3 | ± | 0.1 | |  | 4.7 | ± | 0.1 |
|  | **6-8** |  | 7.7 | ± | 0.5 |  | 5.7 | ± | 0.0 |  | 6.1 | ± | 0.0 |  | 6.0 | ± | 0.2 | |  | 4.8 | ± | 0.1 |

**References**

Andrews S. (2012). FastQC: A quality control tool for high throughput sequence data. Available online at: http://www.bioinformatics.bab.

Ben-Dov E, Brenner A, Kushmaro A. (2007). Quantification of sulfate-reducing bacteria in industrial wastewater, by real-time polymerase chain reaction (PCR) using *dsrA* and *apsA* genes. *Microb Ecol* **54**: 439–451.

Bond DR, Master T, Nesbø CL, Izquierdo-Lopez A V., Collart FL, Lovley DR. (2005). Characterization of citrate synthase from *Geobacter sulfurreducens*  and evidence for a family of citrate synthases similar to those of eukaryotes throughout the *Geobacteraceae*. *Appl Environ Microbiol* **71**: 3858–3865.

Bravo AG, Loizeau JL, Dranguet P, Makri S, Björn E, Ungureanu VG, *et al.* (2016). Persistent Hg contamination and occurrence of Hg-methylating transcript (*hgcA*) downstream of a chlor-alkali plant in the Olt River (Romania). *Environ Sci Pollut Res* 1–13.

Chamberlain SA, Szöcs E. (2013). taxize: taxonomic search and retrieval in R. *F1000Research* **2**: 191.

Eddy SR. (2011). Accelerated profile HMM searches. *PLoS Comput Biol* **7**: e1002195.

Edgar RC. (2004). MUSCLE: Multiple sequence alignment with high accuracy and high throughput. *Nucleic Acids Res* **32**: 1792–1797.

Edgar RC. (2013). UPARSE: highly accurate OTU sequences from microbial amplicon reads. *Nat Methods* **10**: 996–8.

El Fantroussi S, Verschuere L, Verstraete W, Top EM. (1999). Effect of phenylurea herbicides on soil microbial communities estimated by analysis of 16S rRNA gene fingerprints and community-level physiological profiles. *Appl Environ Microbiol* **65**: 982–988.

Fu L, Niu B, Zhu Z, Wu S, Li W. (2012). CD-HIT: Accelerated for clustering the next-generation sequencing data. *Bioinformatics* **28**: 3150–3152.

Joshi NA, Fass JN. (2011). Sickle: A sliding-window, adaptive, quality-based trimming tool for FastQ files.

Martin M. (2011). Cutadapt removes adapter sequences from high-throughput sequencing reads. *EMBnet.journal* **17**: 10–12.

Matsen FA, Kodner RB, Armbrust EV. (2010). pplacer : linear time maximum-likelihood and Bayesian phylogenetic placement of sequences onto a fixed reference tree pplacer : linear time maximum-likelihood and Bayesian phylogenetic placement of sequences onto a fixed reference tree. http://matsen.fhcrc.org/pplacer/pplacer.pdf.

McMurdie PJ, Holmes S. (2014). Waste not, want not: why rarefying microbiome data is inadmissible. *PLoS Comput Biol* **10**: e1003531.

Podar M, Gilmour CC, Brandt CC, Soren A, Brown SD, Crable BR, *et al.* (2015). Global prevalence and distribution of genes and microorganisms involved in mercury methylation. *Sci Adv* **1**: e1500675–e1500675.

Regier N, Frey B, Converse B, Roden E, Grosse-Honebrink A, Bravo AG, *et al.* (2012). Effect of *Elodea nuttallii* roots on bacterial communities and MMHg proportion in a Hg polluted sediment. *PLoS One* **7**: e45565.

Ritz C, Spiess AN. (2008). qpcR: An R package for sigmoidal model selection in quantitative real-time polymerase chain reaction analysis. *Bioinformatics* **24**: 1549–1551.

Schaefer JK, Kronberg R-M, Morel FMM, Skyllberg U. (2014). Detection of a key Hg methylation gene, *hgcA*, in wetland soils. *Environ Microbiol Rep* **6**: 441–447.

Schaefer JK, Yagi J, Reinfelder JR, Ellickson K, Tel-or S, Barkay T. (2004). Role of the bacterial organomercury lyase (MerB) in controlling methylmercury accumulation in mercury-contaminated natural waters. *Environ Sci Technol* **38**: 4304–4311.

Sinclair L, Osman OA, Bertilsson S, Eiler A. (2015). Microbial community composition and diversity via 16S rRNA gene amplicons: Evaluating the Illumina platform. *PLoS One* **10**: e0116955.

Stamatakis A. (2014). RAxML version 8: A tool for phylogenetic analysis and post-analysis of large phylogenies. *Bioinformatics* **30**: 1312–1313.

Steinberg LM, Regan JM. (2008). Phylogenetic comparison of the methanogenic communities from an acidic, oligotrophic fen and an anaerobic digester treating municipal wastewater sludge. *Appl Environ Microbiol* **74**: 6663–6671.

Tichopad A, Dilger M, Schwarz G, Pfaffl M. (2003). Standardized determination of real-time PCR efficiency from a single reaction set-up. *Nucleic Acids Res* **31**: 122–122.
